# Origin of synergistic effects in bicomponent cobalt oxide-platinum catalysts for selective hydrogenation reaction

**DOI:** 10.1038/s41467-019-11970-8

**Published:** 2019-09-13

**Authors:** Jiankang Zhang, Zhe Gao, Sen Wang, Guofu Wang, Xiaofeng Gao, Baiyan Zhang, Shuangfeng Xing, Shichao Zhao, Yong Qin

**Affiliations:** 1 State Key Laboratory of Coal Conversion, Institute of Coal Chemistry, Chinese Academy of Sciences, 27 Taoyuan South Road, 030001 Taiyuan, P.R. China; 2 Center of Materials Science and Optoelectronics Engineering, University of Chinese Academy of Sciences, 100049 Beijing, P.R. China

**Keywords:** Catalysis, Catalyst synthesis, Catalytic mechanisms, Heterogeneous catalysis

## Abstract

The synergistic nature of bicomponent catalysts remains a challenging issue, due to the difficulty in constructing well-defined catalytic systems. Here we study the origin of synergistic effects in CoO_x_-Pt catalysts for selective hydrogenation by designing a series of closely contacted CoO_x_Pt/TiO_2_ and spatially separated CoO_x_/TiO_2_/Pt catalysts by atomic layer deposition (ALD). For CoO_x_/TiO_2_/Pt, CoO_x_ and platinum are separated by the walls of titania nanotubes, and the CoO_x_-Pt intimacy can be precisely tuned. Like CoO_x_Pt/TiO_2_, the CoO_x_/TiO_2_/Pt shows higher selectivity to cinnamyl alcohol than monometallic TiO_2_/Pt, indicating that the CoO_x_-Pt nanoscale intimacy almost has no influence on the selectivity. The enhanced selectivity is ascribed to the increased oxygen vacancy resulting from the promoted hydrogen spillover. Moreover, platinum-oxygen vacancy interfacial sites are identified as the active sites by selectively covering CoO_x_ or platinum by ALD. Our study provides a guide for the understanding of synergistic nature in bicomponent and bifunctional catalysts.

## Abstract (web summary)

The development of catalysts with high activity and selectivity for hydrogenation remains a challenge. Here the authors report cobalt oxide-platinum catalysts with increased oxygen vacancy resulting from the promoted hydrogen spillover with platinum-oxygen vacancies as active sites.

## Introduction

Bicomponent catalysts have received considerably increasing interests in the recent decades due to the enhanced catalytic properties compared with their single-component counterparts, arising from direct contact between the two components, which can be called as synergistic effects^1,2^. However, due to the limitations in controlling the catalyst microstructures and precisely tuning bicomponent intimacy by the traditional methods, it is still a challenging issue to understand the origin of synergistic effects (i.e., short- or long-range interactions) and identify the active sites.

The general belief is that the bicomponents should be as close as possible (i.e., short-range interactions of bicomponents) to promote electron interaction and/or construct interfaces to achieve effective catalysis. In this case, the interaction of a metal oxide promoter with a metal particle is puzzling due to the presence of multiple potential catalytic active sites under the reaction conditions. In contrast, some recent reports revealed the effects of intimacy on the catalyst performance when the bicomponents are separated and tuned at nanoscale and even millimeter-scale distance (i.e., long-range interactions of bicomponents)^3–10^. For example, Coville and coworkers investigated the effect of Ru and Co intimacy on the activity and selectivity of Co catalysts using conventional incipient wetness impregnation in the typical Fischer–Tropsch reaction^5^. Bokhoven and coworkers prepared a series of model catalyst samples with precisely controlled Pt–FeO_*x*_ intimacy at nanoscale distances using the more precise nanolithography technique, and investigated the hydrogen spillover effects on TiO_2_ and Al_2_O_3_ supports^7^. But no reaction (not possible for such structure) is conducted to verify the proposed hydrogen spillover mechanism under the real reaction conditions. Therefore, well-defined bicomponent catalytic systems are highly desirable to unravel the origin of synergistic effects under the real reaction conditions.

Atomic layer deposition (ALD) is a powerful thin-film technique for synthesis of conformal thin films and highly dispersed nanoparticles^11–16^. Many advanced nanocatalysts have been designed and synthesized by ALD in the past decade^17–26^. Herein, we investigate the effects of CoO_*x*_–Pt intimacy on the selective hydrogenation of cinnamaldehyde (CALD) and the hydrogen spillover effects based on CoO_*x*_Pt/TiO_2_ and well-designed spatially separated structures of CoO_*x*_/TiO_2_/Pt catalysts by a highly controllable and reliable ALD approach. Compared with the CoO_*x*_Pt/TiO_2_, the CoO_*x*_/TiO_2_/Pt catalysts exhibit similar catalytic activity and hydrogenation selectivity for the CALD hydrogenation, though the CoO_*x*_–Pt intimacy of the two catalysts is dramatically different. This long-range promoter effect is not broken even when we selectively deposit additional coating layers on the CoO_*x*_ surfaces by ALD. The enhanced selectivity is ascribed to the promoted hydrogen spillover and thus increased oxygen vacancies (O_v_). The well-designed structures can be synthesized and used to study the synergetic natures of other bicomponent and bifunctional catalysts with enhanced performance for other reactions.

## Results

### Synthesis and characterization of the catalysts

**Fig. 1:**
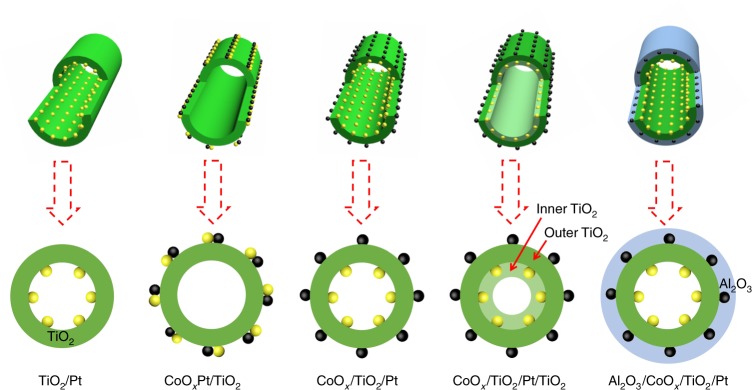
Schematic illustration of the catalysts. Semi-sectional and cross-sectional views of the different catalysts prepared by ALD. The yellow and black balls represent Pt and CoO_*x*_, respectively

The closely contacted CoO_*x*_Pt/TiO_2_ and spatially separated CoO_*x*_/TiO_2_/Pt catalysts were synthesized by a template-assisted ALD strategy (Fig. 1 and Supplementary Scheme 1)^26^. For the synthesis of CoO_*x*_Pt/TiO_2_, an amorphous TiO_2_ film was first deposited on carbon nanocoils (CNCs) used as templates by ALD, obtaining TiO_2_/CNCs, and then Pt nanoparticles were deposited on the TiO_2_/CNCs by Pt ALD, obtaining Pt/TiO_2_/CNCs. Subsequently, the CNCs were removed by calcination under an air atmosphere, producing Pt/TiO_2_ catalysts with anatase nanotubes. Lastly, CoO_*x*_ nanoparticles were deposited on the outer surface of the Pt/TiO_2_ through CoO_*x*_ ALD, producing CoO_*x*_Pt/TiO_2_ catalysts with the closest CoO_*x*_–Pt intimacy (Supplementary Scheme 1a). The CoO_*x*_/TiO_2_/Pt catalysts were prepared by exchanging the deposition sequence of TiO_2_ and Pt (Supplementary Scheme 1b). The CoO_*x*_–Pt intimacy can be precisely controlled by adjusting the thickness of TiO_2_ layer. In addition, the CoO_*x*_/TiO_2_/Pt/TiO_2_ and Al_2_O_3_/CoO_*x*_/TiO_2_/Pt catalysts (Fig. 1) were also prepared by selectively covering Pt with TiO_2_ and CoO_*x*_ nanoparticles with Al_2_O_3_, respectively, to identify the real active sites in the bicomponent catalysts.

**Fig. 2:**
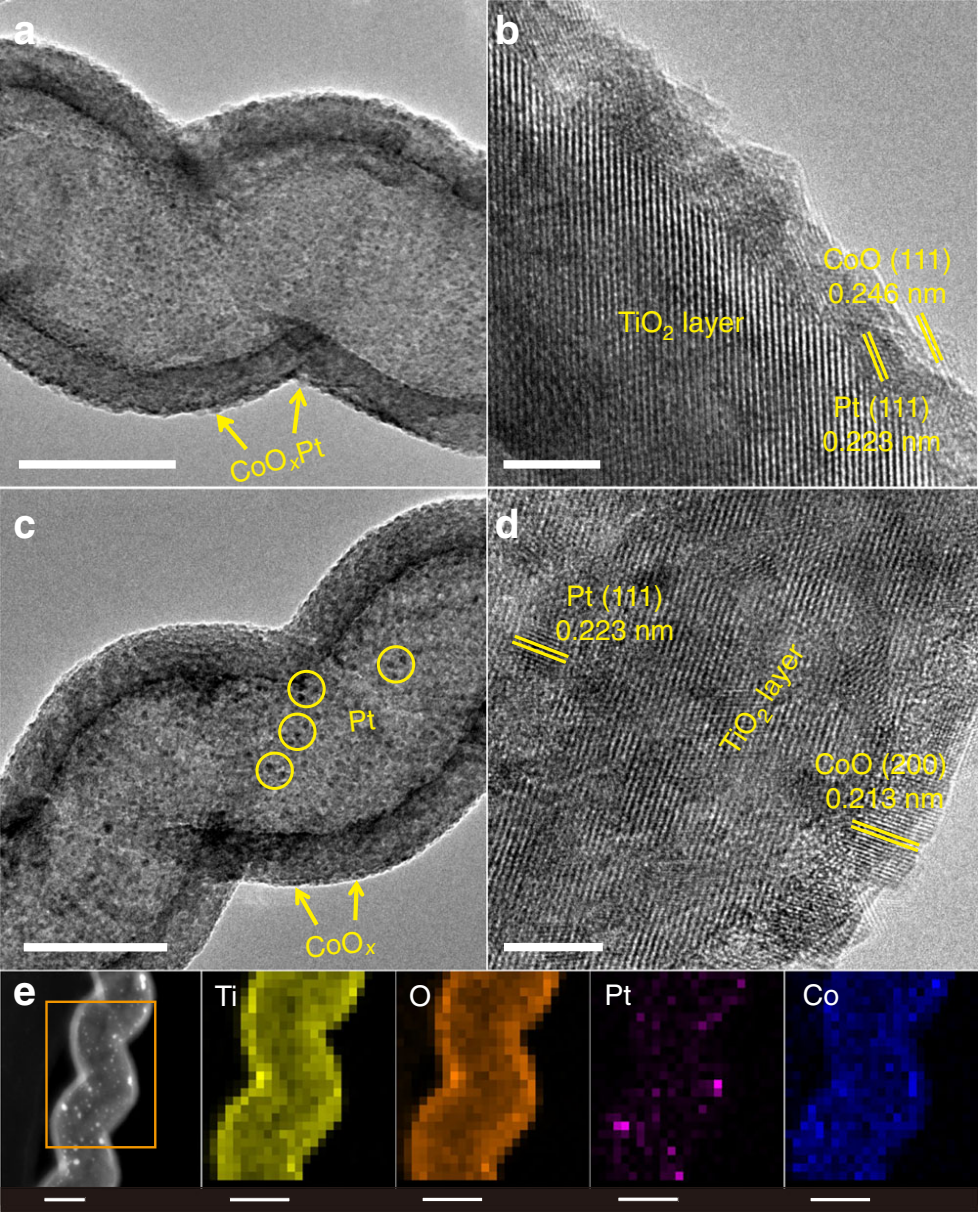
Structural characterizations of the two catalysts. TEM and HRTEM images of the CoO_*x*_Pt/TiO_2_ (**a**, **b**) and CoO_*x*_/TiO_2_/Pt (**c**, **d**) catalysts. **e** STEM image of the CoO_*x*_/TiO_2_/Pt catalysts and the corresponding EDS mapping profiles in rectangular area. Scale bars: **a**, **c** and **e** 50 nm; **b** and **d** 5 nm

Figure 2 shows the transmission electron microscopy (TEM) and high-resolution TEM (HRTEM) images of the CoO_*x*_Pt/TiO_2_ and CoO_*x*_/TiO_2_/Pt catalysts, prepared with 150, 20 and 300 cycles for CoO_x_, Pt and TiO_2_ deposition, respectively. The Pt and CoO_*x*_ nanoparticles, with average sizes of 2.9 nm and 4.3 nm, respectively (Supplementary Figs. 1 and 2), are highly dispersed on the outer surface of the anatase TiO_2_ nanotubes with a wall thickness of ~13.7 nm, in which CoO_*x*_ is deposited on/next to Pt forming CoO_*x*_–Pt interfaces (Fig. 2a, b). The measured lattice distances of the nanoparticles are ~0.223 nm and ~0.246 nm (Fig. 2b), which correspond to the Pt(111) and CoO (111) planes, respectively^27,28^. The larger lattice distance of 0.350 nm matches well with (101) planes of anatase TiO_2_^26^. For the CoO_*x*_/TiO_2_/Pt catalyst (Fig. 2c, d), Pt nanoparticles (indicated by yellow circles) with an average particle size of 3.1 nm (Supplementary Fig. 3) are uniformly deposited on the inner surface of the TiO_2_ nanotubes, while CoO_*x*_ nanoparticles are uniformly deposited on the outer surface of the TiO_2_ nanotubes (Supplementary Fig. 4). In CoO_*x*_Pt/TiO_2_ catalysts, the Pt nanoparticles are closely contacted with CoO_*x*_ nanoparticles, while in CoO_*x*_/TiO_2_/Pt catalysts, the average CoO_*x*_–Pt distance is corresponded to the TiO_2_ thickness. Thus the average CoO_*x*_–Pt distance in CoO_*x*_Pt/TiO_2_ is much smaller than that of CoO_*x*_/TiO_2_/Pt. The sample was further characterized by using high-angle annular dark-field scanning TEM (HAADF-STEM), and energy-dispersive X-ray spectroscopy (EDS) mapping (Fig. 2e and Supplementary Figs. 5 and 6), further confirming that the Pt and CoO_*x*_ nanoparticles are separately decorated on the inner and outer surfaces of the TiO_2_ nanotubes, respectively.

### Catalytic performance

The selective hydrogenation of C=O in α, β-unsaturated aldehydes (e.g., CALD) to value-added unsaturated alcohols has been of increasing interest for the production of fine chemicals and pharmaceutical precursors^29–38^. However, it remains challenging to achieve high-yield unsaturated alcohols since the hydrogenation of C=C is thermodynamically more favorable than that of C=O. To address the issue, various catalysts have been designed and synthesized, and Pt-based catalysts were found to be more desirable in the selective reduction of C=O than other metals^29–38^. Bitter and coworkers found that Pt particle size and oxygen groups on carbon nanofiber supports have a vital influence on the selectivity to cinnamyl alcohol (CALC)^29^. Chen and coworkers reported that Co–Pt/SiO_2_ bimetallic catalysts exhibit better catalytic activity and selectivity than Pt/SiO_2_ and Cu–Pt/SiO_2_ catalysts due to the electronic property modification of Pt^30,31^. Similar results were also reported by Tsang et al., i.e., the selective C=O hydrogenation can be achieved with high activity using Co-decorated Pt nanocrystals as nanocatalysts^38^.

To unravel the synergetic mechanism, the selective hydrogenation of CALD is selected as an example to evaluate the catalytic properties of the as-prepared catalysts, and the results are summarized in Table 1. Note that the Pt nanoparticles are located at different positions for the Pt/TiO_2_ and TiO_2_/Pt catalysts. For the monometallic TiO_2_/Pt catalyst with Pt loading of 3.7 wt.% (Supplementary Table 1), the main product is hydrocinnamaldehyde (HALD) obtained from hydrogenation of the C=C and the selectivity to HALD amounts to 62.5%, while the selectivity to the desired product CALC, obtained from hydrogenation of the C=O, is only 16.2% (Entry 1). For the CoO_*x*_/TiO_2_ reference catalyst, only trace conversion (0.9%) is detected under the same reaction conditions (Entry 2). For the CoO_*x*_Pt/TiO_2_ catalysts with closely contacted Pt and CoO_*x*_, the selectivity to CALC is remarkably improved after addition of CoO_*x*_ (Entry 3). Moreover, the product of excessive hydrogenation to hydrocinnamyl alcohol (HALC) is also obviously suppressed. Similar phenomenon is also found for the CoO_*x*_-supported Pt-based catalysts, and the Pt40/CoO_*x*_ catalysts exhibit the best catalytic performance with 90.2% conversion and 80.3% selectivity to CALC (Supplementary Fig. 7). The improved selectivity to CALC is usually ascribed to the promoter effects, originating from electronic or structural modifications^35–41^. Unexpectedly, similar hydrogenation results are also achieved over the CoO_*x*_/TiO_2_/Pt (13.7 nm, 300-cycle TiO_2_) catalysts with nanoscale CoO_*x*_–Pt intimacy (Entry 4 and Supplementary Fig. 8). Moreover, only slight decrease in hydrogenation activity and selectivity is observed over the CoO_*x*_/TiO_2_(900)/Pt catalysts (Entry 5) when the TiO_2_ layer becomes much thicker (40.5 nm, 900-cycle TiO_2_, Supplementary Figs. 9 and 10).

**Table 1:** Selective hydrogenation results of CALD over the different catalysts^a^

Entry	Catalysts	Conversion (%) (CALD)	Selectivity (%)		
			(CALC)	(HALD)	(HALC)
1	TiO_2_/Pt	93.1	16.2	62.5	21.7
2	CoO_*x*_/TiO_2_	0.9	–	–	–
3	CoO_*x*_Pt/TiO_2_	92.4	82.1	14.5	3.3
4	CoO_*x*_/TiO_2_/Pt	91.3	81.5	13.9	4.3
5	CoO_*x*_/TiO_2_(900)/Pt^b^	89.8	80.3	14.3	4.7
6	CoO_*x*_/TiO_2_/Pt/TiO_2_	8.8	52.1	47.4	–
7	Al_2_O_3_/CoO_*x*_/TiO_2_/Pt^c^	90.2	77.8	15.9	5.8
8	Al_2_O_3_/Pt^d^	31.8	–	100	–
9	CoO_*x*_/Al_2_O_3_/Pt	18.5	39.1	60.7	–
10	CoO_*x*_Pt/Al_2_O_3_	27.4	69.4	30.2	–
11	CoO_*x*_/Al_2_O_3_	0.2	–	–	–

^a^Reaction conditions: 30 mL of 0.8 mmol cinnamaldehyde in ethanol and 2 MPa H_2_ were placed in 50 mL stainless-steel autoclave (65 °C for 1.5 h)

^b^The cycle number of TiO_2_ is 900 for the catalysts. For all other catalysts, the cycle number of TiO_2_ is 300

^c^Dense Al_2_O_3_ layer is used to guarantee the complete covering of CoO_*x*_ species

^d^The cycle number of Al_2_O_3_ is 100 for the Al_2_O_3_-based catalysts (Entries 8–11)

Furthermore, control experiments were also carried out by selectively exposing Pt or CoO_*x*_ to confirm the roles played by CoO_*x*_ and Pt during the reaction. The hydrogenation conversion and selectivity to the desired CALC product decrease remarkably when Pt is selectively covered by two-layered TiO_2_ (CoO_*x*_/TiO_2_/Pt/TiO_2_, Entry 6). When CoO_*x*_ is selectively covered by an outer dense Al_2_O_3_ layer of ~11.5 nm (Al_2_O_3_/CoO_*x*_/TiO_2_/Pt), no visible change is observed in the catalytic performance (Entry 7). These results indicate that Pt–TiO_2_ interface regions should be the main active sites for the CALD hydrogenation reaction, instead of CoO_*x*_. It should be noted that the selectivity of CALC hydrogenation is guaranteed to improve with CoO_*x*_ addition due to the long-range promoter effect and that this effect is not broken even when the surface of CoO_*x*_ promoter is entirely covered by a dense Al_2_O_3_ layer (Al_2_O_3_/CoO_x_/TiO_2_/Pt, Entry 7). This effect is altered when the TiO_2_ layer is changed to Al_2_O_3_ (~11.5 nm), i.e., the catalytic performance is considerably influenced by the CoO_*x*_–Pt intimacy (Entries 8–11). Closely contacted CoO_*x*_Pt/Al_2_O_3_ catalysts exhibit higher activity and selectivity to the desired product than those of the separated CoO_*x*_/Al_2_O_3_/Pt catalysts with nanoscale intimacy. This should be related to the poor capability of spillover hydrogen transfer on the nonreducible Al_2_O_3_^7^. In addition, the reusability of CoO_*x*_/TiO_2_/Pt for selective hydrogenation of CALD was also tested (Supplementary Fig. 11). There is no obvious decrease in the conversion after five catalytic cycles, indicating a good stability of the catalysts.

### X-ray absorption fine structure measurements

**Fig. 3:**
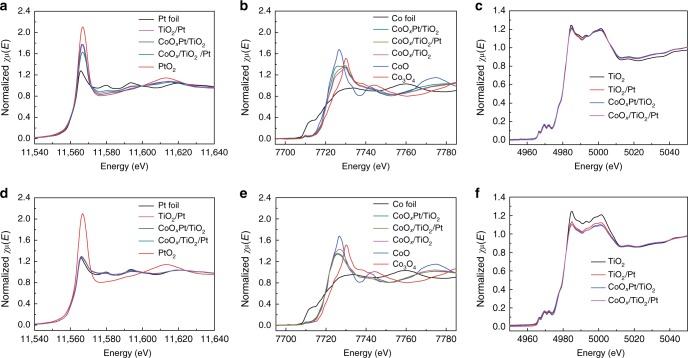
XANES spectra of the catalysts. **a** Ex situ and **d** in situ Pt L_3_-edge XANES spectra of TiO_2_/Pt, CoO_*x*_/TiO_2_/Pt, CoO_*x*_Pt/TiO_2_, and reference Pt foil and PtO_2_; **b** Ex situ and **e** in situ Co K-edge XANES spectra of TiO_2_/Pt, CoO_*x*_/TiO_2_/Pt, CoO_*x*_Pt/TiO_2_, and reference Co foil, CoO and Co_3_O_4_; **c** Ex situ and **f** in situ Ti K-edge XANES spectra of TiO_2_/Pt, CoO_x_Pt/TiO_2_, CoO_x_/TiO_2_/Pt and reference TiO_2_

The electronic states of the TiO_2_/Pt, CoO_*x*_/TiO_2_/Pt and CoO_*x*_Pt/TiO_2_ catalysts before and during the hydrogenation reaction processes were investigated by ex situ and in situ X-ray absorption fine structure measurements (Fig. 3). Figure 3a shows the normalized Pt L_3_-edge ex situ X-ray absorption near-edge structure (XANES) spectra of the as-prepared catalysts, and the reference spectra of Pt foil and PtO_2_. The white line intensity of the as-prepared catalysts falls in the range between the Pt foil and PtO_2_, suggesting that the as-prepared Pt nanoparticles consist of a certain amount of PtO_*x*_ in addition to metallic Pt^0^. Expanded view of the white lines (Supplementary Fig. 12) indicates the different electronic states of Pt for TiO_2_/Pt, CoO_*x*_/TiO_2_/Pt and CoO_*x*_Pt/TiO_2_ catalysts. Figure 3b presents the normalized Co K-edge XANES spectra of the as-prepared catalysts before the reaction, and the reference spectra of Co foil, CoO and Co_3_O_4_. Obviously, the CoO_*x*_/TiO_2_/Pt catalysts also exhibit different white line shapes in comparison with the CoO_*x*_Pt/TiO_2_ catalysts, indicating the different electronic states of the CoO_*x*_ species between the two catalysts. These results demonstrate that before the reaction, the electronic interaction between CoO_*x*_ promoter and Pt should exist (Supplementary Fig. 13). During the reaction process, the PtO_*x*_ species of TiO_2_/Pt, CoO_*x*_/TiO_2_/Pt and CoO_*x*_Pt/TiO_2_ are fully reduced to metallic Pt^0^ due to the presence of H_2_ (Fig. 3d). The Fourier transform spectra of the Pt L_3_-edge extended X-ray absorption fine structure (EXAFS) oscillations for these three catalysts also confirm the full reduction of PtO_*x*_ during the reaction process (Supplementary Fig. 14a, b). For the CoO_*x*_/TiO_2_ reference catalysts, the white line shifts to a low energy during the reaction process (Fig. 3b and e), indicating that the cobalt oxide species at high valence could be reduced to relatively low valence states (e.g., from Co_3_O_4_ to CoO) under a H_2_ atmosphere without Pt assistance (Supplementary Fig. 14c, d). Lower energy shifts of the white lines can be observed for the CoO_*x*_Pt/TiO_2_ and CoO_*x*_/TiO_2_/Pt catalysts during the reaction process, demonstrating that more cobalt oxide species with lower valence states are formed in the two catalysts compared with the CoO_*x*_/TiO_2_ catalysts, which can be ascribed to the presence of Pt contributing to the reduction of CoO_*x*_ due to the hydrogen spillover effect. In addition, the CoO_*x*_Pt/TiO_2_ and CoO_*x*_/TiO_2_/Pt catalysts exhibit almost the same white line shapes during the reaction process (Fig. 3e), i.e., the valence states of the formed cobalt oxide species of the two catalysts are nearly identical; this is due to the strong reduction ability of the spillover hydrogen (H·), generated on Pt, toward CoO_*x*_, which is nearly independent of the CoO_*x*_–Pt intimacy on reducible TiO_2_ supports in the nanoscale range^7^.

Figure 3c and f shows the normalized Ti K-edge ex situ and in situ XANES spectra of TiO_2_/Pt, CoO_*x*_/TiO_2_/Pt, CoO_*x*_Pt/TiO_2_ and the reference spectra of TiO_2_. Before the reaction, the Ti K-edge white line curves of all the three catalysts coincide, indicating the same electronic states of their TiO_2_ supports. During the hydrogenation reaction conditions, in situ XANES spectra show reduction of Ti^4+^ to Ti^3+^ for all three catalysts, and CoO_x_/TiO_2_/Pt and CoO_*x*_Pt/TiO_2_ possess more reduced Ti^3+^ than that of the TiO_2_/Pt catalyst. The k^3^-weighted EXAFS spectra of the three catalysts and the reference TiO_2_, and their fitted curves are presented in Supplementary Fig. 15. The fitted results (Supplementary Table 2) are consistent with the XANES results.

### Chemisorption

**Fig. 4:**
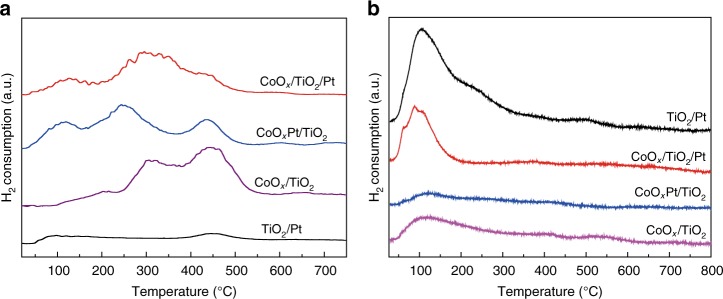
Chemisorption characterizations. **a** H_2_-TPR and **b** H_2_-TPD profiles of the catalysts

Figure 4a shows the H_2_ temperature programmed reduction (H_2_-TPR) profiles of the catalysts. From the H_2_-TPR profiles of the CoO_*x*_Pt/TiO_2_, CoO_*x*_/TiO_2_/Pt and CoO_*x*_/TiO_2_ catalysts (also see Supplementary Fig. 10), both a low temperature (110–220 °C, Co_3_O_4_ to CoO) and a high temperature (245–345 °C, CoO to Co^0^) reduction peak can be observed. No peaks related to platinum oxides are observed, probably because the amounts of H_2_ used for the reduction of PtO_x_ species in the catalysts are beyond the lower detection limits of the detector. In addition, the reduction peak at higher temperature (~450 °C) could be assigned to the partial reduction of TiO_2_, indicating that TiO_2_ is more difficult to reduce^42^. Compared with the CoO_*x*_/TiO_2_ catalysts, the temperatures needed for CoO_*x*_ reduction are much lower for the CoO_*x*_Pt/TiO_2_ and CoO_*x*_/TiO_2_/Pt catalysts due to the presence of Pt, further revealing the effect of hydrogen spillover from Pt to CoO_*x*_^31^. Note that the reduction temperature needed for the CoO_*x*_Pt/TiO_2_ catalyst is slightly lower than that of the CoO_*x*_/TiO_2_/Pt catalyst due to the closer CoO_*x*_–Pt intimacy (Fig. 4a). Figure 4b shows the H_2_ temperature programmed desorption (H_2_-TPD) profiles of the catalysts. The TiO_2_/Pt and CoO_*x*_/TiO_2_/Pt catalysts exhibit a much higher H_2_ adsorption ability than that of the CoO_*x*_/TiO_2_ and CoO_*x*_Pt/TiO_2_ catalysts, indicating that Pt atoms are the adsorption and dissociation sites of H_2_, which is further verified by the following density functional theory (DFT) simulation.

## Discussion

**Fig. 5:**
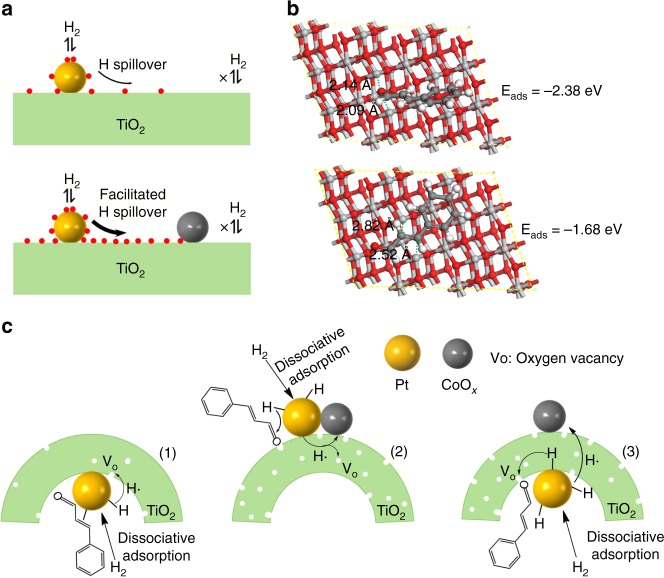
DFT calculation and proposed reaction mechanisms. **a** The effect mechanism of CoO_*x*_ on the hydrogen spillover over TiO_2_-supported Pt catalysts. **b** The optimized structure for adsorption of CALD molecule using C=O (top) or C=C (bottom) double bond on the O_v_ of TiO_2_. Atom coloring: Ti, gray; O, red; H, white; C, black. **c** The possible enhancement mechanism for selective CALD hydrogenation reaction. (1), (2) and (3) represent TiO_2_/Pt, CoO_*x*_Pt/TiO_2_ and CoO_*x*_/TiO_2_/Pt catalysts, respectively. For TiO_2_/Pt, the number of O_v_ is limited, leading to its low selectivity. For CoO_*x*_Pt/TiO_2_ and CoO_*x*_/TiO_2_/Pt, the addition of CoO_*x*_ promotes the formation of O_v_ sites through hydrogen spillover and thus improves the selectivity, regardless of whether CoO_*x*_ and Pt are separated by a TiO_2_ layer or not

In this work, we discovered a hydrogen spillover effect for the CALD hydrogenation reactions. Hydrogen spillover is a reversible and dynamic equilibrium process. Modulation of the spillover conditions, such as temperature and gas partial pressure, hinders or facilitates the spillover process and can be used for controlling the oxide reduction^7,43^. Generally, the activation and dissociation of H_2_ molecules on Pt is quite easy and generally considered to be barrierless^7,43–45^, i.e., the energy needed to activate H_2_ molecules on Pt is much lower than that on CoO_*x*_ and TiO_2_ (Supplementary Fig. 16). For the TiO_2_/Pt catalyst under the reaction conditions, hydrogen spillover occurs (Fig. 5a). Active hydrogen species migrate from Pt onto TiO_2_ supports, leading to the partial reduction of TiO_2_ supports (Ti^4+^ is reduced to Ti^3+^) with the formation of O_v_ around the interface regions^7,46–50^. When CoO_*x*_ is added, in situ XAFS results show that CoO_*x*_ can be reduced to lower valent states through hydrogen spillover under the reaction conditions. More hydrogen species are consumed by CoO_*x*_, thus the original equilibrium of hydrogen spillover is disturbed (Fig. 5a). The generation of active hydrogen species and their transfer process (from Pt to CoO_*x*_ directly, or via TiO_2_ supports) are promoted, until new equilibrium is reached. The consumption of hydrogen species can be seen as the pumps for the migration of atomic hydrogen from Pt toward TiO_2_. The enhanced hydrogen spillover facilitates the reduction of TiO_2_ supports, which is proved by in situ Ti K-edge XANES spectra (Fig. 3c and f), accompanying the increased formation of O_v_ sites.

The adsorption energies of different adsorption configurations of a CALD molecule on the O_v_ formed on TiO_2_ were calculated (Fig. 5b). The calculated adsorption energy of the C=O bond adsorbed on the oxygen vacancies is −2.38 eV, which is larger than that of the C=C bond on the oxygen vacancies (−1.68 eV), indicating that the adsorption configurations of the CALD molecule through the C=O bond on the O_v_ are more stable.

Based on the above discussions, the possible enhancement mechanism is proposed. For the TiO_2_/Pt catalyst, the number of O_v_ at the Pt–TiO_2_ interface is limited under the reaction conditions, leading to its low selectivity (Fig. 5c). When CoO_*x*_ promoters are added, the selectivity to CALC is improved. The promotion can be ascribed to the CoO_*x*_ promoter-induced increase of O_v_ sites through hydrogen spillover effect. For CoO_*x*_/TiO_2_/Pt catalysts, although CoO_*x*_ is separated from the Pt catalyst by a TiO_2_ layer, this long-range CoO_*x*_ promoter can still enhance the catalytic selectivity to CALC. XAFS results demonstrate that the electronic states of CoO_*x*_ of CoO_*x*_Pt/TiO_2_ and CoO_*x*_/TiO_2_/Pt catalysts are almost the same during the reaction process, meaning that the active hydrogen species generated on Pt nanoparticles are still consumed by CoO_*x*_, though CoO_*x*_ is far from Pt. The energy barrier for the transfer of an electron and a proton among the different TiO_2_ sites can be easily overcome under the reaction conditions^7^, and thus the distance of hydrogen spillover across TiO_2_ layer almost has no influence on the catalytic performance. The addition of CoO_*x*_ results in the increased generation of O_v_ sites on the Pt–TiO_2_ interface regions and the remarkably improved selectivity to CALC.

To further clarify the long-range promoter effect (i.e., the role of CoO_*x*_ played), we selectively deposit additional oxide layers on the surfaces of CoO_*x*_ or Pt by ALD. From the XAFS results, it can be concluded that CoO_*x*_ species in the catalysts can be reduced in two ways. Firstly, the CoO_*x*_ species can be reduced under H_2_ atmosphere; secondly, the CoO_*x*_ species can be further reduced to a lower valence state by the active H species spilled from Pt nanoparticles through TiO_2_ support. When Pt is selectively covered by TiO_2_ (CoO_*x*_/TiO_2_/Pt/TiO_2_), the access of CALD molecules to Pt is nearly blocked due to the diffusion limitation (Supplementary Figs. 17 and 18, Supplementary Table 3), leading to the decreased catalytic performance (Entry 6, Table 1). On the contrary, when CoO_*x*_ is selectively covered by a dense Al_2_O_3_ layer (Al_2_O_3_/CoO_*x*_/TiO_2_/Pt), the first way is blocked (Supplementary Fig. 18), while the second way is still preserved and CALD molecules can still have access to Pt via open the ends of the TiO_2_ nanotubes (with a diameter of ~70–90 nm), leading to a nearly unchanged catalytic performance (Entry 7, Table 1). These results further demonstrate that H spillover way is the main path to improve selectivity, and that Pt–O_v_ interface instead of CoO_*x*_ should be the active site for the CALD hydrogenation reaction.

In summary, we have successfully developed a general method based on template-assisted ALD to synthesize closely contacted CoO_*x*_Pt/TiO_2_ and spatially separated CoO_*x*_/TiO_2_/Pt catalysts, achieving the precise tuning of CoO_*x*_–Pt intimacy by varying the deposition sequence and the wall thickness of the TiO_2_ nanotubes. We discover that hydrogen spillover effect generated by the addition of CoO_*x*_ can cause the increase in O_v_, providing the adsorption sites for CALD via C=O bond and correspondingly resulting in the enhancement of selectivity to CALC in the CALD hydrogenation reactions. This hydrogen spillover effect is not broken even when we selectively deposit additional oxide layers on the CoO_*x*_ promoter by ALD to cover its surface entirely. Our work has demonstrated an efficient strategy based on ALD for the design of various bicomponent catalysts with distinct promoter–metal intimacy and the assembly of metals and oxide supports, which is helpful to reveal the origin of bicomponent synergy, the enhancement mechanism and the real active sites.

## Methods

### Synthesis of CNCs and the ALD process

The detailed CNC synthesis process and the ALD process can be found in our previous reports^24–26^.

### Synthesis of TiO_2_/Pt catalysts

CNCs were firstly decorated with Pt nanoparticles by Pt ALD (20 cycles) and then coated with a TiO_2_ layer (300 cycles) by TiO_2_ ALD, producing TiO_2_/Pt/CNCs. After the ALD processes, TiO_2_/Pt/CNCs were calcinated at 500 °C for 2 h in air to remove the CNC templates, obtaining TiO_2_/Pt catalysts, in which Pt nanoparticles were confined in TiO_2_ nanotubes.

### Synthesis of CoO_*x*_/TiO_2_/Pt catalysts

The above-obtained TiO_2_/Pt catalysts were then deposited with cobalt oxides (denoted as CoO_*x*_) by CoO_*x*_ ALD (150 cycles), obtaining shell-isolated CoO_*x*_/TiO_2_/Pt catalysts.

### Synthesis of CoO_*x*_Pt/TiO_2_ catalysts

CNCs were firstly coated with a TiO_2_ amorphous film by TiO_2_ ALD, and then Pt nanoparticles were deposited on the TiO_2_/CNCs by Pt ALD obtaining Pt/TiO_2_/CNCs composites. Subsequently, CNCs were removed by calcination under an air atmosphere producing Pt/TiO_2_ catalysts with porous anatase nanotubes. Lastly, CoO_*x*_ nanoparticles were deposited on the outer surface of Pt/TiO_2_ through CoO_*x*_ ALD, producing CoO_*x*_Pt/TiO_2_ catalysts with the closest CoO_*x*_–Pt intimacy.

### Synthesis of CoO_*x*_/TiO_2_/Pt/TiO_2_ catalysts

CNCs were first coated with a TiO_2_ layer (150 cycles) by TiO_2_ ALD and then decorated with Pt nanoparticles by Pt ALD and another TiO_2_ layer (300 cycles) producing TiO_2_/Pt/TiO_2_/CNCs. After the ALD processes, TiO_2_/Pt/TiO_2_/CNCs were calcinated at 500 °C for 2 h in air to remove the CNC templates, obtaining sandwich-like TiO_2_/Pt/TiO_2_ catalysts, in which Pt nanoparticles are coated by the two-layer TiO_2_. Lastly, the obtained TiO_2_/Pt/TiO_2_ catalysts were decorated with CoO_*x*_ nanoparticles by CoO_*x*_ ALD producing CoO_x_/TiO_2_/Pt/TiO_2_.

### Synthesis of Al_2_O_3_/CoO_*x*_/TiO_2_/Pt catalysts

CNCs were sequentially decorated with Pt nanoparticles by CoO_*x*_ ALD, coated with a TiO_2_ layer (300 cycles) by TiO_2_ ALD, decorated with Pt nanoparticles by Pt ALD and then coated with an Al_2_O_3_ layer (100 cycles) by Al_2_O_3_ ALD producing Al_2_O_3_/CoO_*x*_/TiO_2_/Pt/CNCs, which were calcinated at 550 °C for 2 h in air to remove the CNC templates obtaining Al_2_O_3_/CoO_*x*_/TiO_2_/Pt catalysts, in which CoO_*x*_ nanoparticles were covered by the outer Al_2_O_3_ layer.

### Catalyst characterizations

TEM and HRTEM images were taken with a JEOL-2100F field-emission transmission electron microscope operated at 200 kV. HAADF-STEM images and EDS mapping profiles were collected on a JEOL ARM-200F field-emission transmission electron microscope operated at 200 kV. The X-ray diffraction (XRD) patterns were recorded by using a Philips X’Pert Pro Super X-ray diffractometer with Cu Kα radiation (*λ* = 1.540 Å) in the 2*θ* range from 10° to 90°. The X-ray photoelectron spectra (XPS) were collected on an ESCALab-250 X-ray photoelectron spectrometer with an Al Kα source (1486.6 eV). The XANES and EXAFS spectra of the Pt L_3_-edge and Co K-edge were measured on the BL14W1 beamline of the Shanghai Synchrotron Radiation Facility (SSRF), Shanghai Institute of Applied Physics (SINAP), China, operated at 3.5 GeV. A Si (111) double-crystal monochromator was used to reduce the harmonic component of the monochrome beam. Pt foil, PtO_2_, Co foil, CoO and Co_3_O_4_ were used as reference samples and measured in the transmission mode, and all the catalysts were also measured in the transmission mode. The diffuse reflectance infrared Fourier transform spectroscopy (DRIFTS, CO chemisorption) measurements were performed on a Bruker Vector 22 spectrometer. After the sample was loaded, it was firstly reduced by 10%H_2_/Ar at 150 °C for 1.5 h. Then the sample was cooled to 30 °C under 10%H_2_/Ar atmosphere, on which a background spectrum was collected. Subsequently, CO was introduced onto the sample until saturation, followed by Ar purge to remove the physically adsorbed gaseous CO. Finally, CO-DRIFTS spectra were collected with 250 scans. H_2_-TPR experiments were performed in a tubular quartz reactor (TP-5080, Tianjin Xianquan, China), into which 50 mg sample was loaded. The reduction was conducted in a 10% H_2_/N_2_ atmosphere at a heating rate of 10 °C/min. H_2_-TPD experiments were performed in the same apparatus. A 50-mg sample was firstly reduced in situ at 250 °C for 1 h in a 10% H_2_/N_2_ flow and then cooled to 30 °C in the same atmosphere. Subsequently, the sample was swept with nitrogen at a flow rate of 30 sccm for 30 min to remove physisorbed or weakly bound species. TPD was performed by heating the sample from room temperature to 800 °C at a ramp rate of 10 °C/min in nitrogen. N_2_ adsorption–desorption experiments were performed on a BELSORP-Mini system at 77 K. The specific surface area was determined using the Brunauer–Emmett–Teller (BET) method, and the pore size distributions were calculated by the Barrett–Joyner–Halenda (BJH) method according to the desorption branches. The Pt and Co metal content of the samples was determined by inductively coupled plasma mass spectrometry (ICP-MS) analysis (Thermo ICAP 6300).

### Catalytic activity measurements

CALD hydrogenation reactions were performed on the above catalysts in a 50-mL stainless-steel autoclave reactor. The reaction was carried out at 65 °C and 2.0 MPa H_2_ with a certain amount of catalysts in 30 mL of ethanol, and 100 μL of CALD. After the reaction, the reactor was cooled and then slowly depressurized. Finally, the reaction mixture was separated by centrifugation in order to remove the solid catalysts. The reaction products were analyzed and quantified by gas chromatographic mass spectrometry (GC-MS, Agilent, 7890A). The reaction conversion and selectivity were determined by the product analysis.

### Computational method

Periodic DFT calculations within the generalized gradient approximation (GGA) were conducted with the Vienna ab initio Simulation Package (VASP 5.3.5) with considering the spin-polarization, in which a projector-augmented potential (PAW) method is implemented. The Perdew–Burke–Ernzerhof (PBE) functional at the GGA level was used, and the plane wave basis set was cut off at the energy of 400 eV. For our ALD-prepared cobalt-based catalysts, the obtained cobalt oxides consist of Co_3_O_4_ and CoO and the main species is CoO. Therefore, CoO was selected for DFT calculations. The CoO surface was modeled with a six GGA extension atomic layer 3 × 3 CoO(111) slab. DFT + U corrections (an effective onsite Coulomb interaction parameter) were employed to mitigate the self-interaction errors of Co 3d orbital with U-J of 7.1 eV. The main exposed crystal plane for ALD-prepared TiO_2_ is the thermodynamically most stable (101) surface, and thus (101) surface was chosen as a model for the anatase TiO_2_ interface. The TiO_2_ surface was modeled with a six atomic layer 3 × 3 slab. The slab and its image were separated by a vacuum region of 15 Å. The reactant molecular and the top two layers of these model systems were relaxed, whereas other atoms were fixed in their initial lattice sizes.

## Supplementary information

Supplementary Information

## Data Availability

All the relevant data are available from the authors upon request.
