# Supplementary material for: Origin of synergistic effects in bicomponent cobalt oxide-platinum catalysts for selective hydrogenation reaction
Source: Nat Commun. 2019 Sep 13;10:4166. doi: 10.1038/s41467-019-11970-8 (PMC6744570; doi:10.1038/s41467-019-11970-8)
Supplement: Supplementary file 1 — Supplementary Information [file 41467_2019_11970_MOESM1_ESM.pdf]

Origin of synergistic effects in bicomponent cobalt oxide platinum catalysts for selective hydrogenation reaction

Zhang et al.

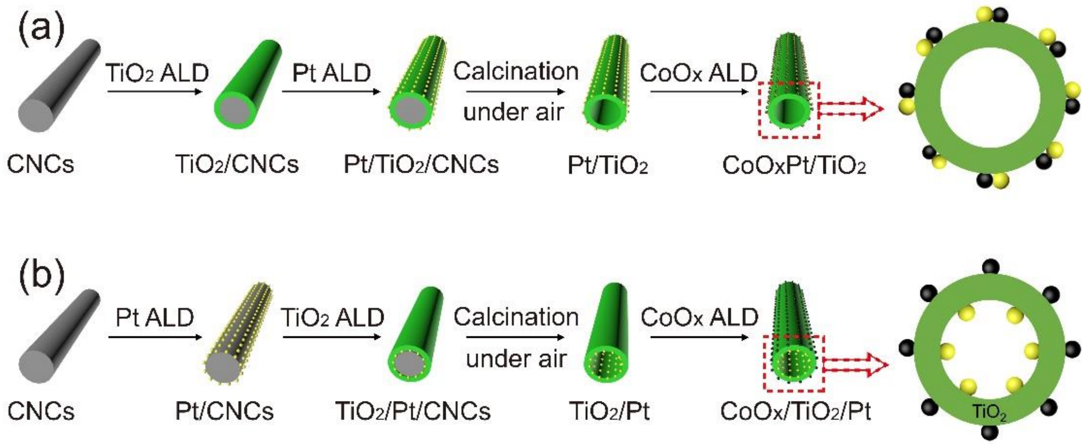

Supplementary Scheme 1. Schematic illustration of the synthesis process of the catalysts. (**a**): CoO_xPt/TiO_2 catalyst; (**b**) $CoO_x/TiO_2/Pt$ catalyst.

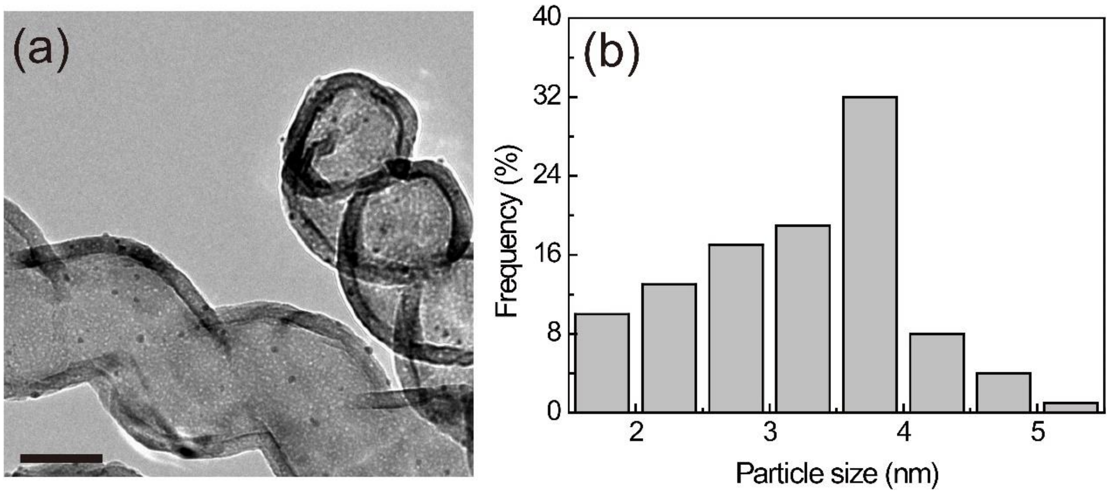

Supplementary Figure 1. TEM characterization of the Pt/TiO_2 catalyst. (**a**) TEM image and the corresponding particle size distribution (**b**) of the Pt/TiO_2 catalysts (The ALD cycle number of Pt is 20). Scale bar of (**a**): 50 nm.

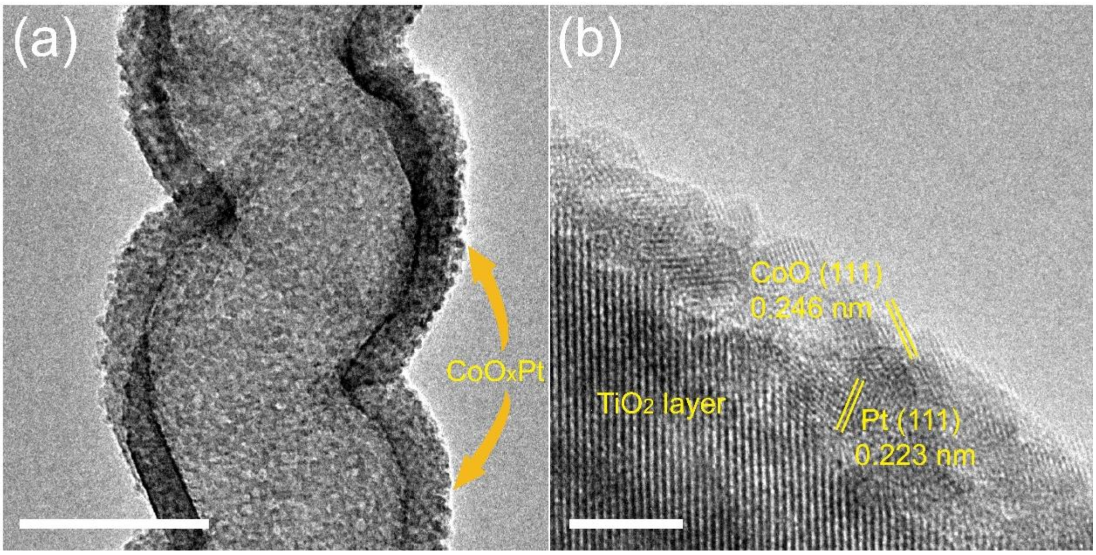

Supplementary Figure 2. TEM characterization of the CoO_xPt/TiO_2 catalyst. (**a**) TEM (**b**) and HRTEM images of the CoO_xPt/TiO_2 catalysts. The ALD cycle numbers for Pt, CoO_x and TiO_2 are 20, 150 and 300 respectively for the catalysts used in our present work unless otherwise indicated. The CoO_x nanoparticles with size of about 4.3 nm form a connected nanoparticle film closely contacting with Pt. Scale bar of (**a**): 50 nm and (**b**): 5 nm, respectively.

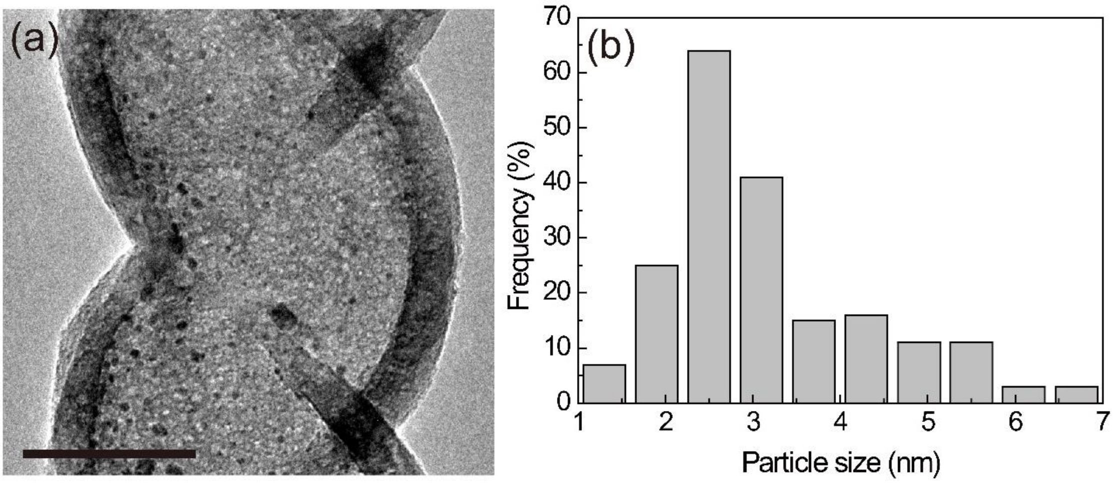

Supplementary Figure 3. TEM characterization of the TiO_2/Pt catalyst. (**a**) TEM image and (**b**) the corresponding particle size distribution of the TiO_2/Pt catalysts (The ALD cycle number of Pt is 20). Scale bar of (**a**): 50 nm.

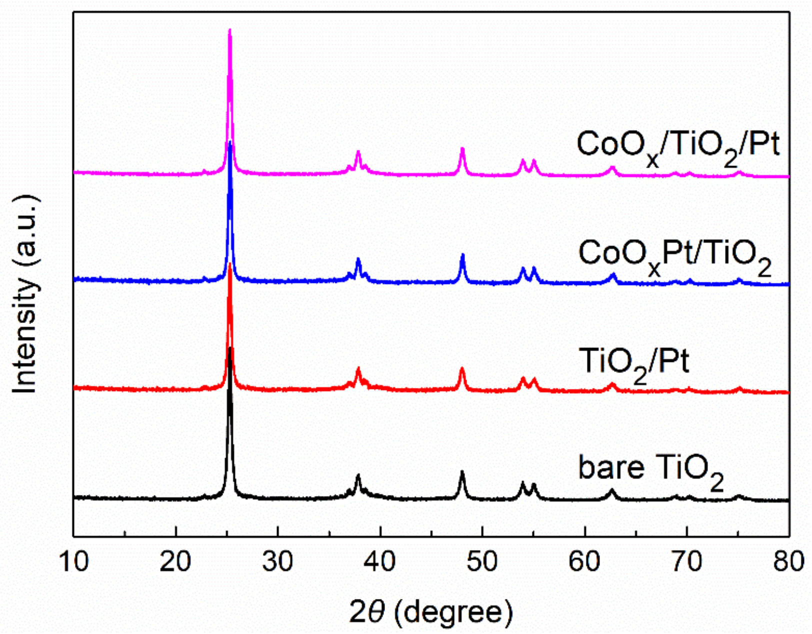

Supplementary Figure 4. XRD patterns of the catalysts. As shown in Supplementary Figure 4, a strong peak at around 25.2° and several weak peaks can be observed, which can be well indexed to anatase TiO_2 (JCPDS No.21-1272). While no peaks assigned to Pt or CoO_x are detected, which can be ascribed to the high dispersion ALD-prepared nanoparticles.

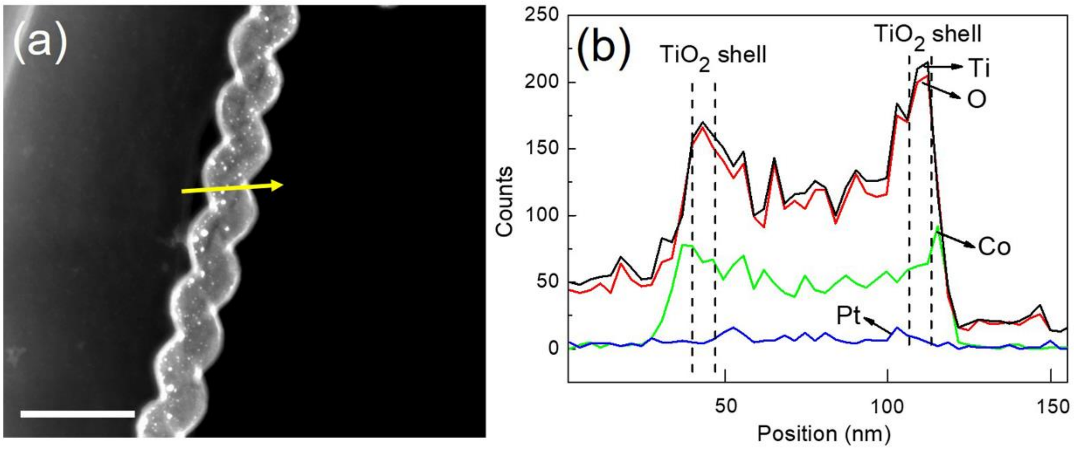

Supplementary Figure 5. Structural characterization of the $CoO_x/TiO_2/Pt$ catalysts. (**a**) HAADF-STEM image and (**b**) EDS line scan of the $CoO_x/TiO_2/Pt$ sample. The different positions of Ti, O, Pt and Co from the EDS line scan of $CoO_x/TiO_2/Pt$ sample reveals that Pt and CoO_x nanoparticles are distributed on the inner and outer surface of the TiO_2 shell, respectively. Scale bar of (**a**): 200 nm.

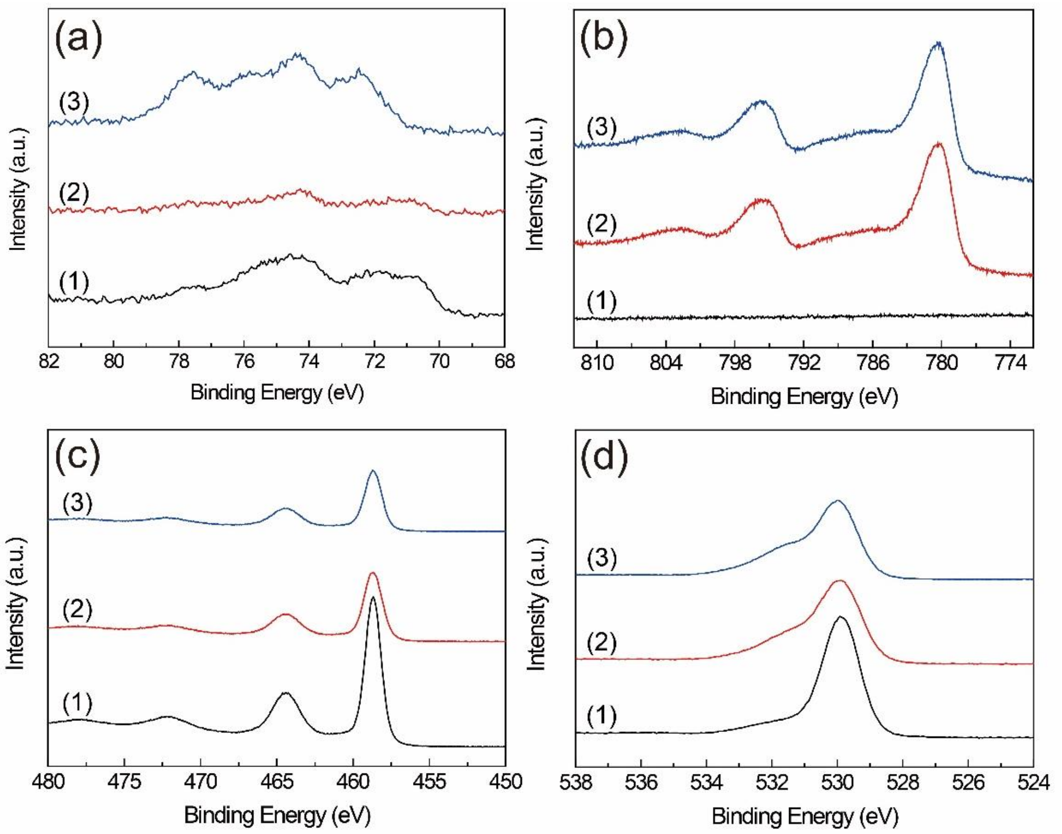

Supplementary Figure 6. XPS characterization of the catalysts. XPS spectra of (**a**) Pt, (**b**) Co, (**c**) Ti and (**d**) O of the three catalysts: (1) TiO_2/Pt, (2) CoO_x/TiO_2/Pt and CoO_xPt/TiO_2. Pt, Co, Ti and O were all observed, again confirming the successful deposition of Pt and CoO_x on TiO_2.

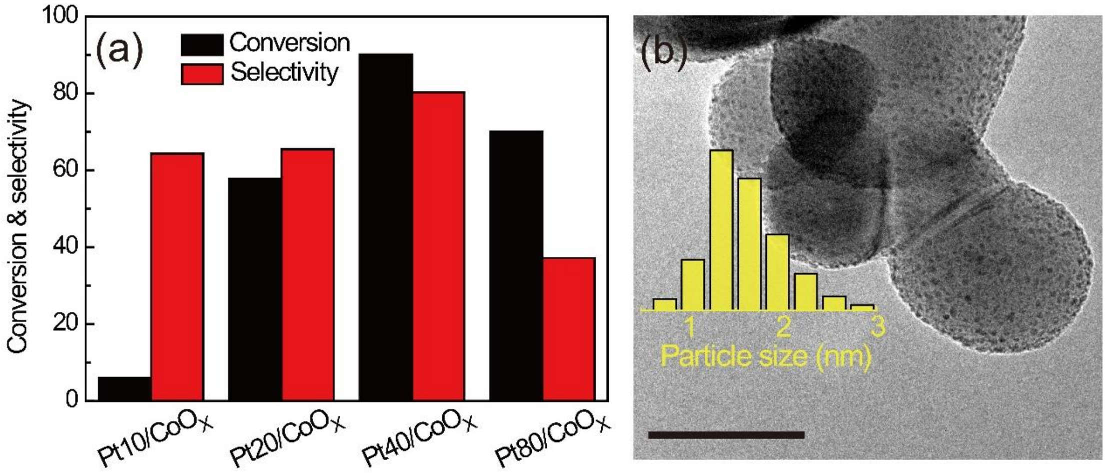

Supplementary Figure 7. Hydrogenation properties and TEM characterization of Pt/CoO_x. (**a**) The selective hydrogenation properties of CALD over the Pt/CoO_x catalysts. (**b**) TEM image of Pt40/CoO_x catalysts and the corresponding histograms (inset) of particle size distribution of Pt (The average particle size is 1.6 nm). Reaction conditions: 30 mL of 0.8 mmol cinnamaldehyde in ethanol, 45 mg catalysts and 2 MPa H_2 were placed in 50 mL stainless steel autoclave (65 °C for 1.5 h). Scale bar of (**b**): 50 nm.

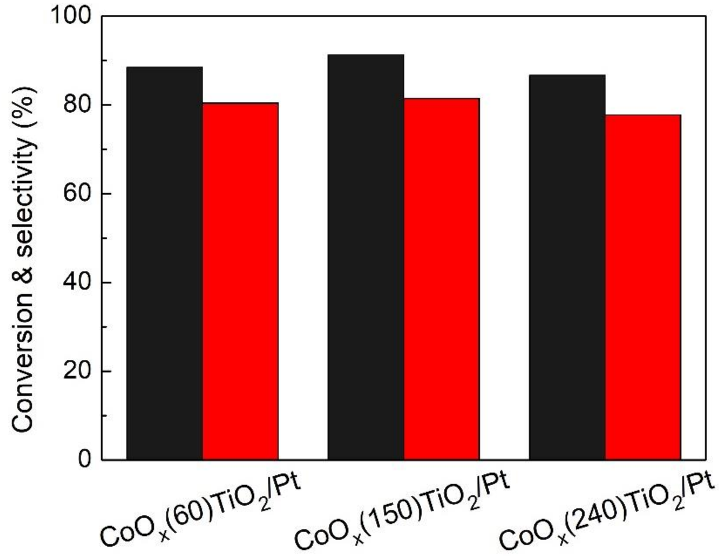

Supplementary Figure 8. Hydrogenation properties of the $CoO_x/TiO_2/Pt$ catalysts. The catalytic activity and selectivity to CALC over $CoO_x/TiO_2/Pt$ catalysts with different CoO_x cycles (i.e., Pt/Co ratios). The black and red bar represent the conversion of CALD and selectivity to CALC. Reaction conditions: 30 mL of 0.8 mmol cinnamaldehyde in ethanol, 20 mg catalysts and 2 MPa H_2 were placed in 50 mL stainless steel autoclave (65 °C for 1.5 h). There is a slight decrease in view of the CALD conversion and selectivity to target product CALC when CoO_x ALD cycle number is 60 compared with the $CoO_x(150)/TiO_2/Pt$ catalysts, while the catalytic activity also decreases slightly when the CoO_x ALD cycle number is increased to 240, indicating that overmuch CoO_x promoter dosage is not beneficial for the enhancement of catalytic performance. The influence of Pt/Co ratios on the catalytic performance may be due to the particle size effects and/or the Pt-Co interactions. To guarantee the close contact between CoO_x and Pt, 150-cycle CoO_x were deposited on Pt/TiO_2 in our present work to form a CoO_x nanoparticle film (i.e., CoO_x-Pt nanoparticles with the closest/atomic-scale intimacy).

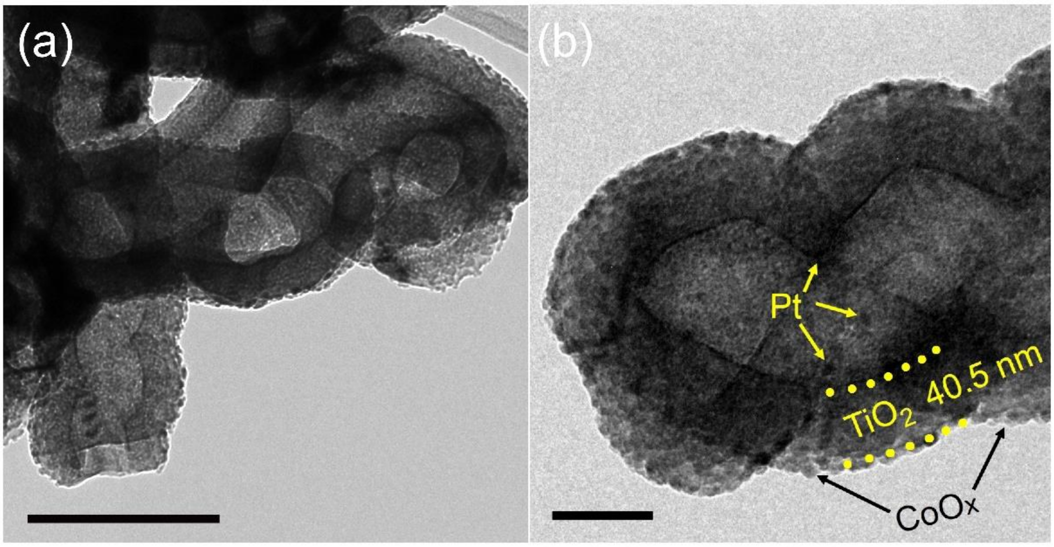

Supplementary Figure 9. TEM characterization of the $CoO_x/TiO_2(900)/Pt$ catalyst. (**a**) Low and (**b**) high magnification TEM images of the $CoO_x/TiO_2(900)/Pt$ catalysts (900 cycles of TiO_2 ALD). Scale bar of (**a**): 200 nm; (**b**): 50 nm.

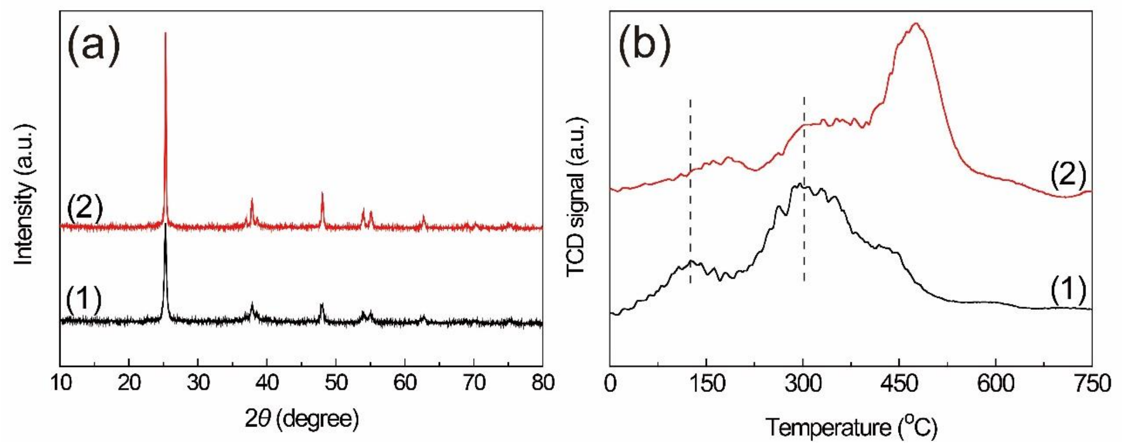

Supplementary Figure 10. Structural characterizations of the $CoO_x/TiO_2/Pt$ catalysts. XRD patterns (**a**) and H_2-TPR profiles (**b**) of the catalysts: (1) $CoO_x/TiO_2(300)/Pt$ and (2) $CoO_x/TiO_2(900)/Pt$.

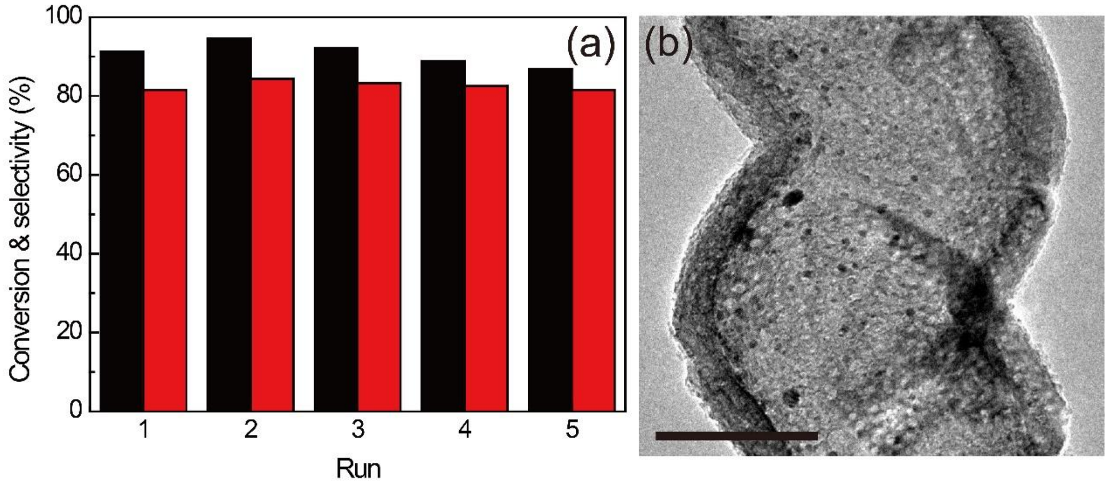

Supplementary Figure 11. The stability of the $CoO_x/TiO_2/Pt$ catalysts. (**a**) The stability tests of the $CoO_x/TiO_2/Pt$ catalysts for the CALD hydrogenation, and (**b**) TEM image of the used catalysts after five catalytic cycles (The black and red bar represent the conversion of CALD and selectivity to CALC). Reaction conditions: 30 mL of 0.8 mmol cinnamaldehyde in ethanol, 20 mg catalysts and 2 MPa H_2 were placed in 50 mL stainless steel autoclave (65 °C for 1.5 h). Scale bar of (**b**): 50 nm.

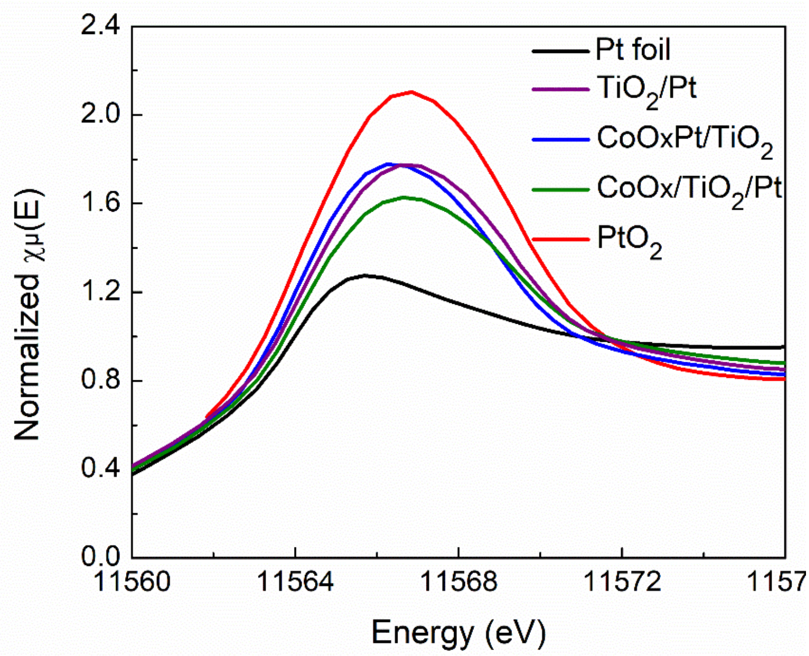

Supplementary Figure 12. XANES analysis of different samples. Expand view of white lines in normalized XANES of the TiO_2/Pt, CoO_xPt/TiO_2, CoO_x/TiO_2/Pt and reference samples.

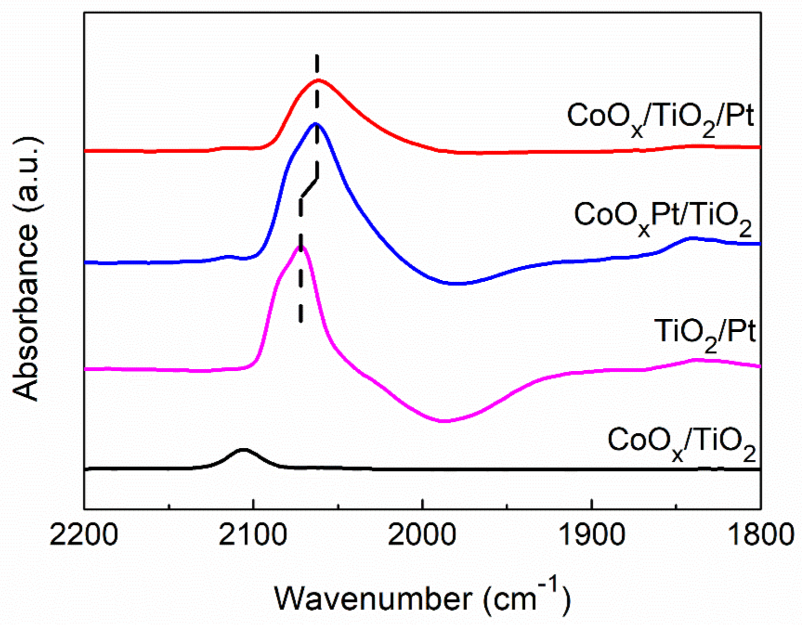

Supplementary Figure 13. CO-DRIFTS spectra of the $CoO_x/TiO_2/Pt$, CoO_xPt/TiO_2, TiO_2/Pt and CoO_x/TiO_2 catalysts.

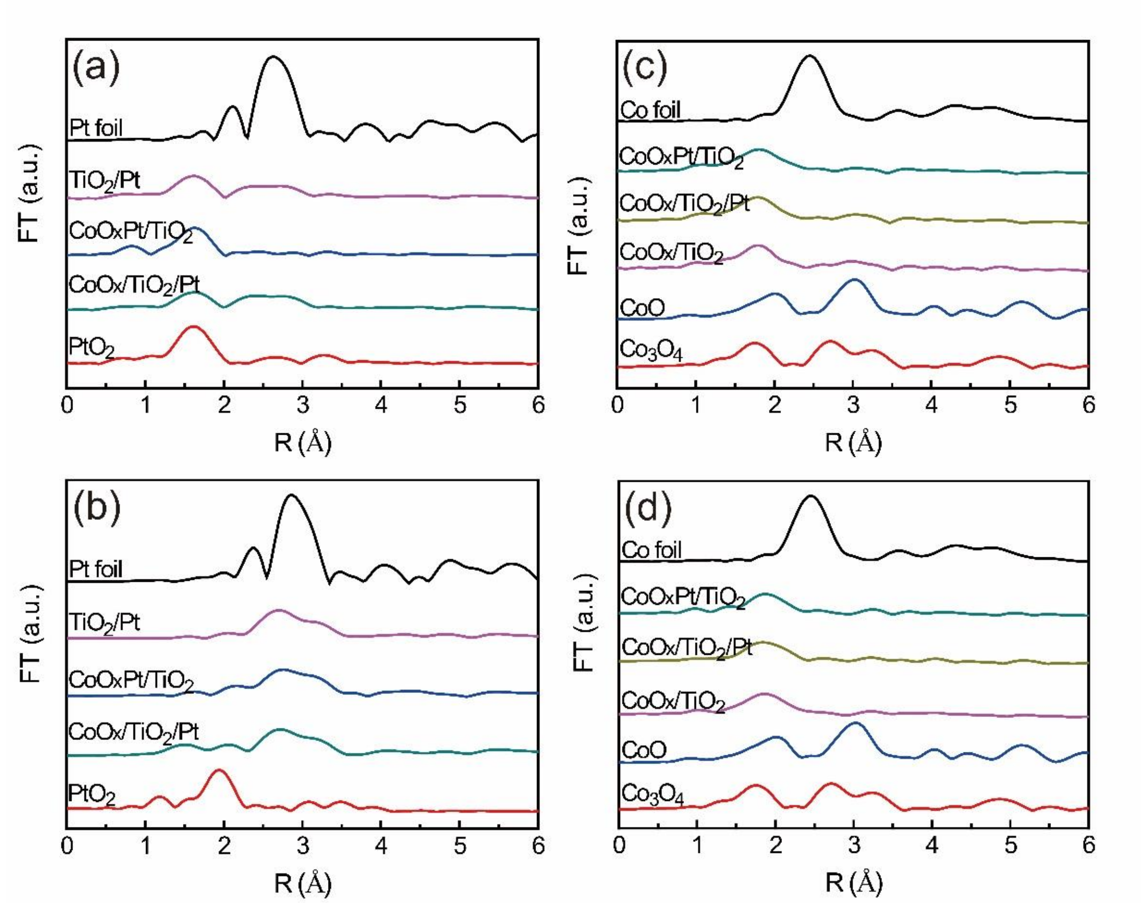

Supplementary Figure 14. EXAFS spectra of the catalysts. (**a**) Ex situ and (**b**) in situ Pt L_3-edge Fourier transform k^3-weighted EXAFS spectra of Pt foil, TiO_2/Pt, CoO_x/TiO_2/Pt, CoO_xPt/TiO_2 and PtO_2. (**c**) Ex situ and (**d**) in situ Co K-edge Fourier transform k^2-weighted EXAFS spectra of Co foil, TiO_2/Pt, CoO_x/TiO_2/Pt, CoO_xPt/TiO_2, CoO and Co_3O_4.

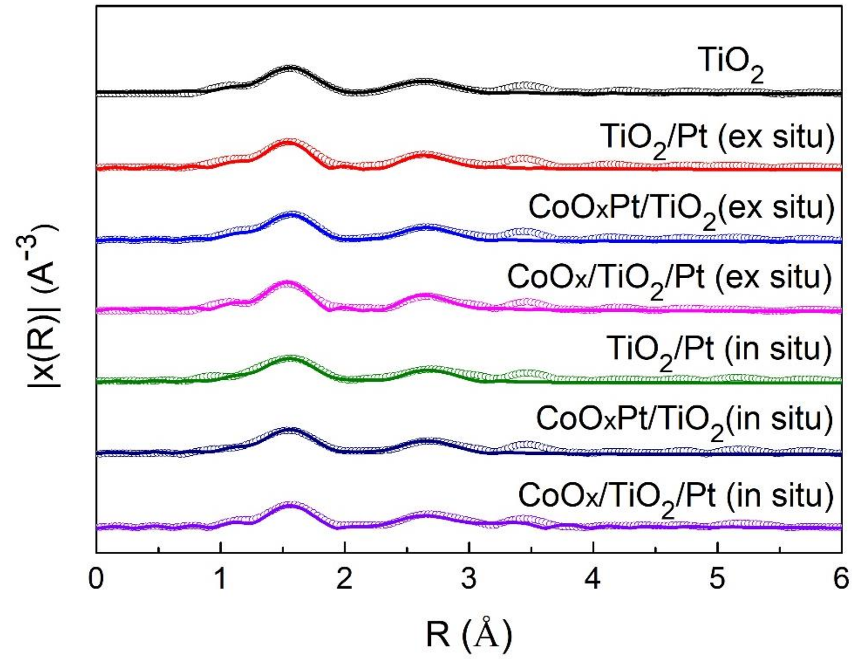

Supplementary Figure 15. EXAFS spectra of different catalysts. Ex situ and in situ Fourier transform k^3-weighted EXAFS spectra of TiO_2, TiO_2/Pt, CoO_x/TiO_2/Pt and CoO_xPt/TiO_2.

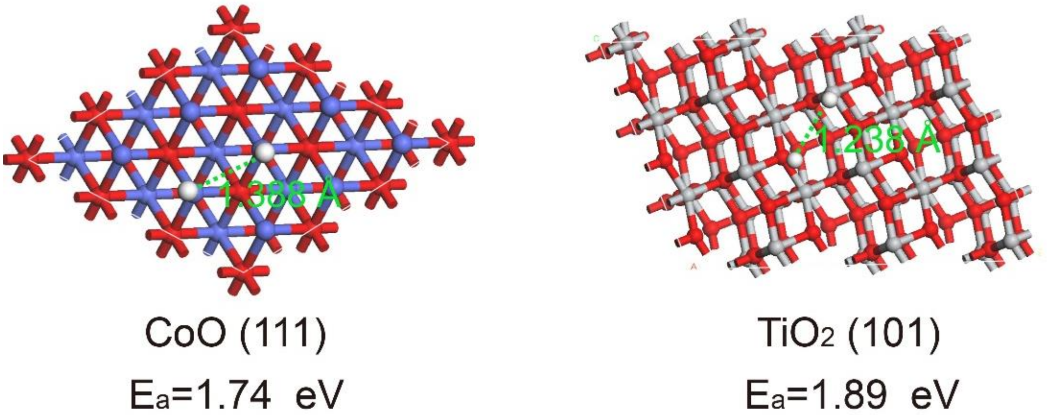

Supplementary Figure 16. The calculated activation energy of H_2 on different sites based on the DFT. CoO (111) and TiO_2 (101) surfaces are chosen as the model active sites to calculate the H_2 activation capability on CoO_x and TiO_2, respectively. The calculated energy needed to activate H_2 molecules (E_a) on CoO (111) and TiO_2 (101) surface are 1.74 and 1.89 eV, respectively, indicating that H_2 molecules can be relatively easily dissociated on CoO (CoO_x).

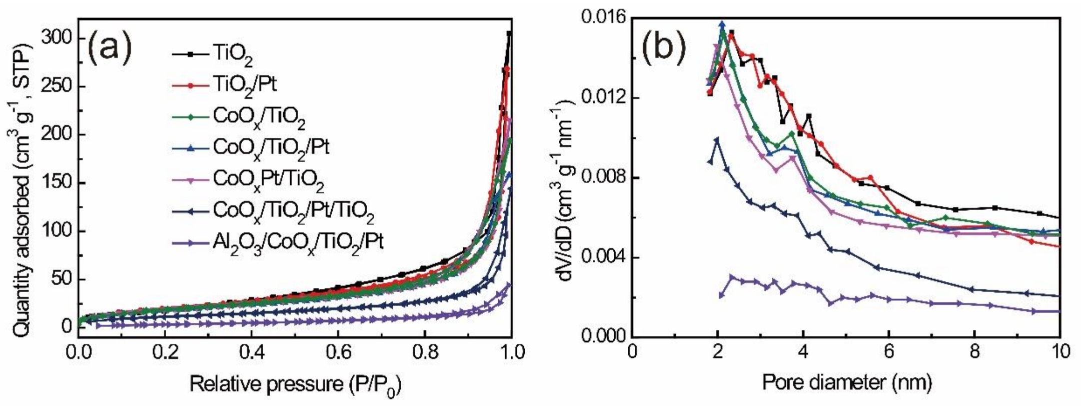

Supplementary Figure 17. Structure characterizations of the catalysts. (**a**) The N_2 adsorption-desorption isotherms and (**b**) and the corresponding pore size distributions of the catalysts.

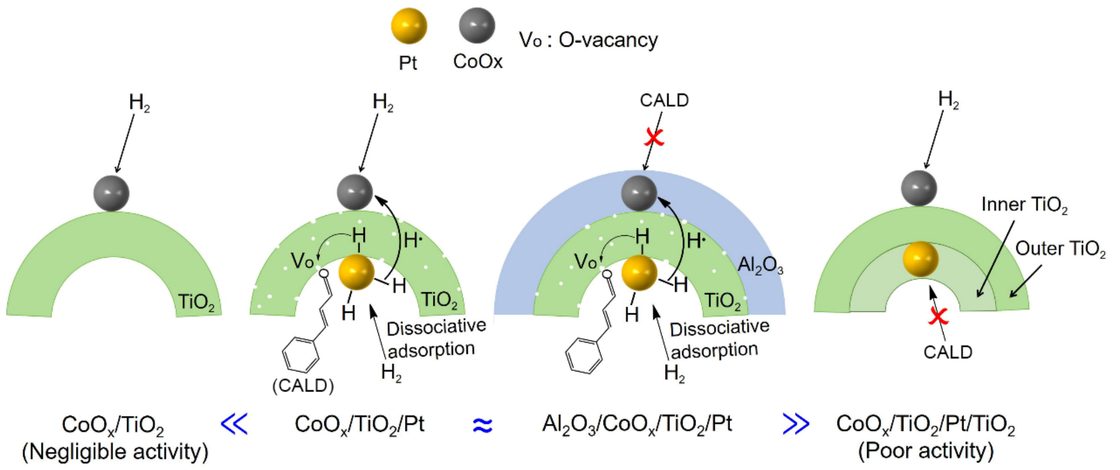

Supplementary Figure 18. The proposed reaction mechanism. The yellow and black balls represent Pt and CoO_X, respectively, and the white dots represent the formed oxygen vacancy (V_O).

Compared with the closely contact CoO_XPt/TiO2 catalysts, the separated CoO_X/TiO2/Pt and even selectively covered Al2O3/CoO_X/TiO2/Pt catalysts also exhibit similar selectivity to CALC, indicating that the remote CoO_X-Pt synergy can also enhance the catalytic performance. When CoO_X is selectively covered by a dense Al2O3 layer (Al2O3/CoO_X/TiO2/Pt), the CALD molecules cannot access to CoO_X in this case, but the CALD and H2 molecules can access to Pt through the open ends of TiO2 nanotubes (with diameter of 70-90 nm). Therefore, the catalytic performance is well preserved compared with CoO_X/TiO2/Pt. However, the Pt is selectively covered by a two-layered porous TiO2 (CoO_X/TiO2/Pt/TiO2), the catalytic performance decreases remarkably, and this is because that the CALD molecules is difficult to access to Pt due to the small size of pores (about 2 nm) greatly increasing the diffusion resistance.

Supplementary Table 1. Loading amount of Pt and Co of the catalysts.

Sample[a]	Pt content (wt.%)	Co content (wt.%)
TiO_2/Pt	3.8	-
CoO_xPt/TiO_2	3.7	5.5
CoO_x/TiO_2/Pt	3.7	5.1

Supplementary Table 2. FT-EXAFS parameters of the samples.

Sample	Shell	R (Å)	CN
TiO_2	Ti-O	1.93	6
	Ti-Ti	3.02	4
Pt/TiO_2 (ex-situ)	Ti-O	1.93±0.02	5.2±0.5
	Ti-Ti	3.01±0.02	3.6±0.4
Pt/TiO_2 (in-situ)	Ti-O	1.94±0.02	4±0.6
	Ti-Ti	3.04±0.02	3.2±0.4
$PtCoO_x/TiO_2$ (ex-situ)	Ti-O	1.93±0.02	5.2±0.6
	Ti-Ti	3.03±0.02	3.7±0.5
$PtCoOx/TiO_2$ (in-situ)	Ti-O	1.95±0.02	4±0.5
	Ti-Ti	3.02±0.02	3.4±0.4
$Pt/TiO_2/CoO_x$ (ex-situ)	Ti-O	1.92±0.02	5.4±0.6
	Ti-Ti	3.02±0.02	3.8±0.4
$Pt/TiO_2/CoO_x$ (in-situ)	Ti-O	1.95±0.02	3.8±0.5
	Ti-Ti	3.01±0.02	3.4±0.4

CN, coordination number; R, bonding distance.

Supplementary Table 3. The specific surface area and pore structure parameters of the catalysts.

Entry	Catalysts	S_{BET} ($m^2\ g^{-1}$)	V_{total} ($cm^3\ g^{-1}$)	D (nm)
1	TiO_2	76.8	0.59	2.3
2	TiO_2/Pt	72.5	0.44	2.3
3	CoO_x/TiO_2	68.6	0.31	2.1
4	CoO_xPt/TiO_2	68.8	0.34	2.0
5	CoO_x/TiO_2/Pt	66.3	0.26	2.1
6	CoO_x/TiO_2/Pt/TiO_2	41.0	0.22	2.0
7	Al_2O_3/CoO_x/TiO_2/Pt	12.9	0.07	--

The average pore diameter (D) generated on TiO_2 nanotubes is about 2.3 nm, and this is slightly decreased after decoration of metals (Entry 1-6) except that no D value is given for the selectively covered Al_2O_3/CoO_x/TiO_2/Pt catalysts due to the very low adsorption amount of N_2 (i.e., the dense Al_2O_3 layer results in the complete cover of CoO_x species and pores of TiO_2 nanotubes). Note that the pore sizes are much lower than diameter of the nanotubes (70-90 nm). That is to say, the CALD molecules may be difficult to diffuse through the pores, while the diffusion of CALD molecules is not influenced through the larger open ends of TiO_2 nanotubes.
